# Enhancing Elderly Nutrition: A Qualitative Evaluation of Menus in a Social Solidarity Institution in the North of Portugal

**DOI:** 10.3390/nu16050753

**Published:** 2024-03-06

**Authors:** Sandra Celina Fernandes Fonseca, Suzanne Carvalho Barroso, Maria Cristina Teixeira Santos

**Affiliations:** 1 Department of Sports, Exercise and Health Sciences, University of Trás-os-Montes e Alto Douro, 5000-801 Vila Real, Portugal; suzanne13barroso@hotmail.com; 2 Research Center in Sports Sciences, Health Sciences and Human Development (CIDESD), 5001-801 Vila Real, Portugal; 3 Faculty of Nutrition and Food Sciences, University of Porto, 4150-180 Porto, Portugal; cristinasantos@fcna.up.pt; 4 ProNutri Group—CINTESIS@RISE, Center for Health Technology and Services Research (CINTESIS), Associate Laboratory RISE—Health Research Network, University of Porto, 4200-450 Porto, Portugal

**Keywords:** elderly, menus, nursing homes, private institutions of social solidarity, qualitative evaluation

## Abstract

This work addresses the importance of food and nutrition in promoting the health of the elderly population, with a specific focus on the qualitative evaluation of menus provided by a social solidarity institution in Portugal. The aim of this study is to conduct a qualitative evaluation of menus furnished by a social solidarity institution situated in the northern region of Portugal in order to prevent and/or treat malnutrition in the elderly. The methodology involves the evaluation of four weekly menus, totaling 28 complete daily menus for the elderly, using the “Avaliação Qualitativa de Ementas Destinadas a Idosos” (AQEDI) tool. This assessment tool comprises six domains: general items, soup, protein suppliers, carbohydrate suppliers, vegetable suppliers, and dessert, each consisting of various parameters. The findings reveal that all menus were classified as “acceptable,” with percentages ranging from 60.73% to 68.84%, and suggest that there exists room for improvement. This study emphasizes the necessity for coordinated efforts within the institution to enhance menu planning, taking into account both nutritional guidelines and sensory aspects of food. Effective coordination within the institution is crucial for maintaining positive aspects and rectifying inadequacies in menu planning.

## 1. Introduction

The aging of the population is occurring within a context of significant social, cultural, economic, institutional, and familial changes. According to Eurostat and the Portuguese Institute of Statistics (INE), it is estimated that the number of institutionalized elderly individuals will increase in the coming years [1,2]. So, the need for care for the elderly brings into focus elderly care institutions and their fundamental role in organizing food services, as well as in assessing and monitoring the nutritional status of its users.

Nutrition plays a fundamental role across all age groups, but its significance is notably amplified concerning the health and well-being of the elderly [3]. It is evident that concerns regarding dietary practices and their impact on health are progressively emerging in care facilities catering to the elderly, including nursing homes, assisted living facilities, and senior care institutions.

Adequate food and nutrition play a crucial role in promoting a healthy aging process, resulting in improved functional capacity and reduced vulnerability to diseases [4,5]. Therefore, continuous monitoring of food intake by healthcare professionals is essential. This involves identifying potential risks or existing nutritional deficiencies and tailoring dietary plans to meet the specific needs of each elderly individual. The responsibility for nutritional intervention spans across various disciplines and requires collaborative efforts. This intervention encompasses different meal preparation methods, food safety practices, and the inclusion of culturally and ethnically appropriate foods, among others. It also aims to address dietary offerings considering the nutritional needs and culinary preferences of a population group with a higher susceptibility to malnutrition [6,7,8,9].

It becomes important to optimize the services provided by these institutions, which are often the sole source of food support for the elderly. It is therefore crucial to ensure that the food provided by these institutions is nutritionally adequate, as well as to address food safety issues. Menu planning should ensure a balanced, varied, and nutritionally rich diet, respecting the sociocultural context of the clients it serves; ensuring a diverse diet that meets the needs of each client, including special dietary requirements and hydration care; considering the preferences of each client; being developed with the collaboration of all responsible parties in the establishment for this process and based on the advice of a nutritionist; being developed, at minimum, on a weekly basis [10,11].

The qualitative assessment of menus for the elderly serves as a crucial mechanism for improving their well-being and overall quality of life, ensuring that their nutritional and dietary needs are adequately met.

Following a study conducted at a social solidarity institution in the municipality of Vila Real [12] that revealed concerning results, with 18.1% of the elderly being malnourished and 45.5% at risk of malnutrition, the objective of the present investigation was to conduct a qualitative evaluation of the menus provided at the institution in question. The purpose was to understand the reality and, based on the evidence, be able to intervene by correcting, adjusting, and optimizing the food offered to the elderly.

## 2. Materials and Methods

This study evaluated four weekly menus from the year 2022, totaling 28 complete daily menus adapted for elderly individuals in a social solidarity institution with 80 institutionalized elderly individuals, with an average age of 87 years (maximum age: 107 years and minimum age of 65), comprising 31 men and 49 women. Prevalent pathologies among the institutionalized elderly in this care facility include hypertension, respiratory and cardiac failure, Alzheimer’s disease, Parkinson’s disease, and other degenerative dementias. Among the elderly, 55 were autonomous, 21 required assistance with feeding, and 4 individuals required parenteral nutrition.

Data collection was conducted by the same individuals.

For the evaluation of the menus, a qualitative evaluation of menus for the elderly, the AQEDI tool (Avaliação Qualitativa de Ementas Destinadas a Idosos), was used [10]. The AQEDI tool facilitates a comprehensive post-cooking analysis and enables a comparison between planned and executed menus. Additionally, it allows for the identification of necessary modifications before menu execution, allowing for preemptive corrections [10].

This qualitative menu evaluation grid takes into account various nutritional and dietary recommendations, some specifically targeting the population aged 65 or older and others aimed at the general population. This tool integrates criteria drawn from existing assessments and introduces novel parameters based on the Dietary Approaches to Stop Hypertension (DASH) diet recommendations [10].

This grid is specifically structured into six evaluation domains (general items, soup, protein suppliers, carbohydrate suppliers, vegetable suppliers, and dessert), with parameters quantified based on their relative importance for ensuring menu quality, totaling 43. Each domain is assigned a specific weight in percentage. Each criterion is assigned a relative importance score based on its necessity, recommendation, or desirability for a healthy diet, translating to the following scoring: 3 = required criterion; 2 = recommended criterion; 1 = desirable criterion.

Upon evaluation, if the menu fulfills the assessed criterion, the corresponding score is assigned; otherwise, a score of zero is assigned. The menu’s overall score is then converted into a percentage using the following formula, consistent with other evaluation grids [8].

Final Score (%) = [(S1 × 0.5) × 100]/MScore1 + [(S2 × 0.1) × 100]/MScore2 + [(S3 × 0.1) × 100]/MScore3 + [(S4 × 0.1) × 100]/MScore4 + [(S5 × 0.1) × 100]/MScore5 + [(S6 × 0.1) × 100]/MScore6

S = Sum of the values obtained in each domain.

MScore = Value of the maximum possible scores in each domain. (In the first domain, if the answer to item 1.16 is positive, MScore = 60; if it is negative, then MScore = 53; in the second domain, MScore = 8; in the third domain, MScore = 15; in the fourth domain, MScore = 5; in the fifth domain, MScore = 4; and in the sixth domain, MScore = 7).

To convert the percentage obtained from the quantitative evaluation into a qualitative assessment, the following criteria were applied—Less than 50%: Not Acceptable; 50% to less than 75%: Acceptable; 75% to less than 90%: Good; 90% to 100%: Very Good.

These criteria allow for a qualitative interpretation of the quantitative scores obtained from the evaluation process.

## 3. Results

Based on the percentage values provided for Menus 1, 2, 3, and 4, which are 60.73%, 68.84%, 62.31%, and 63.95%, respectively, all of these menus fall within the “acceptable” range according to the qualitative assessment criteria.

Table 1 presents the results regarding the compliance and non-compliance of each item, the quantitative outcome per domain, and the final quantitative and qualitative assessment of the menu.

## 4. Discussion

Due to the scarcity of studies focused on menus for the elderly, the discussion of these results is somewhat limited. It is important to note that different published studies utilize various evaluation tools, which occasionally hindered a direct comparison with the results of this study.

Upon completing the evaluation grid, it was observed that in the “General Items” domain, technical sheets for each menu were not provided, which is a method recommended by Lima [13] to convey nutritional information about the meal. Technical specifications for menus are crucial as they offer detailed information about ingredients, preparation methods, and the nutritional content of the dishes. These documents aid in consistent and efficient meal planning and preparation, as well as provide valuable information to users with dietary restrictions or concerns. Moreover, food specification sheets are vital for financial management, assisting in determining the cost of dishes and controlling expenses related to selected ingredients.

Regarding the “General Items,” it is noteworthy that each evaluated menu only includes four daily meals instead of the recommended five [10]. Recommendations suggest three main meals and two or three minor meals of good nutritional quality daily to distribute daily intakes of energy and nutrients over many meals, since metabolic capacity for digestion and absorption is likely to decrease with increasing age. Meals and snacks played various roles as contributors to total energy and nutrient content [14].

Regarding the qualitative evaluation of the soup, all the menus received an acceptable score primarily due to the variety of vegetable products. This finding aligns with a study conducted by Guerra and Rocha [15] in 45 preschool children, where it was observed that there was only one weekly meal of “canja” (chicken soup) in all evaluated menus. Compliance with the recommendation of using legumes as a base in the soup 2 to 3 times a week at a minimum also contributed to the positive result in the “Soup” item, consistent with the study conducted by Cardoso [16] in an evaluation of three daily meals of 45 elderly individuals, where soup typically consisted of vegetables and/or legumes. This contrasts with the study conducted by Lopes and Rocha [17], an evaluation of school menus in the municipality of Pombal, Portugal, where the result was negative due to the scarce use of legumes in the soup. The daily presence of soup cooked with vegetable products, which are excellent sources of vitamins, minerals, and fiber, in the menu is one of the recommendations for a healthy diet [18]. Soup and water provide fluid with a hydrating effect on our bodies, along with sparkling water, flavored water, hot or cold tea, coffee, milk and milky drinks, fruit juices, sports or soft drinks, and smoothies [19]. Drinks should be chosen according to the preferences of the older person, as well as the drinks’ fluid and nutritional content, ensuring that milky drinks, fruit juices, and smoothies, high-calorie drinks, and fortified drinks all have particular benefits in specific circumstances. Observational data have suggested that the number of drinks offered to older adults in residential care is strongly positively associated with fluid intake [11,20,21].

Adequate water intake is also an extremely important factor in the diets of older adults, especially considering that hydration needs to increase during the summer. It is important to note that older adults may have a diminished thirst reflex, making them more prone to dehydration [11].

Concerning “Protein Suppliers,” it is noteworthy that there is an equitable offering between fish and meat dishes. However, the provision of dishes using eggs as the main protein source falls below recommendations in all the evaluated menus, with eggs being used only in two menus once a week. Dietary recommendations from the Institute of Medicine, as disclosed by McGuire [22], suggest the inclusion of an egg dish in the menu plan with a minimum frequency of once a week. A similar result was found in a study conducted by Reis, Figueiredo, and Ávila [23], which highlighted the lack of weekly egg availability. Eggs are a rich source of high-biological-value proteins and predominantly monounsaturated and polyunsaturated fats. They are also excellent providers of minerals (phosphorus, iron, and zinc) and vitamins A, B complex, and D [24]. According to national recommendations, the emphasis should be on offering “white meats” instead of “red meats” [18]. All evaluated menus complied with the recommendation regarding both “red meats” (maximum of two times a week) and “white meats” (minimum of three times a week). Lima and Rocha [25], on the other hand, found opposite results, with non-compliance regarding “red meats” and compliance regarding “white meats.”

Regarding the recommendation to include fatty fish in the menus at least twice a week, only one of the menus did not meet this parameter. However, the fatty fish used exclusively included salmon, sardines, and tuna. As for lean fish such as monkfish, hake, and cod, they are frequently used, which is beneficial for the elderly due to their ease of digestion [26]. Diet can contribute to successful aging through the consumption of omega-3 fatty acids present in fish and can directly influence the innate immunity process [27].

All dishes in the evaluated menus in this study included a carbohydrate-containing side dish. However, contrary to the study by Lima and Rocha [25], there was an equitable distribution between rice, pasta, and potatoes. Vegetables, due to their nutritional characteristics, are a fundamental component of daily food intake, and their inclusion should be ensured in all meals [18]. It was found that this recommendation was not met in 3 out of the 4 evaluated menus. Similarly, a low availability of vegetables was observed in the works of Guerra and Rocha [15] and Lima and Rocha [25]. The recommendation to offer legumes on the plate at least twice a week was always respected, not as a substitution for the carbohydrate source but as a complement, contrary to what is mentioned in the studies conducted by various authors [15,25,28].

Older patients often suffer from gastrointestinal problems, including constipation and diarrhea. Since dietary fiber may contribute to the normalization of bowel functions, and intake is usually low in geriatric patients, the importance of an adequate intake of dietary fiber is emphasized and can be ensured in the menus evaluated by offering daily soup, vegetables, and fruit as dessert [11].

It was found that seasonal fruit was offered as dessert in all the menus, 10 out of 14 times per week, which is consistent with the study on the quality of menus in various institutions conducted by Lima and Rocha [25], where it was also observed that fruit is the predominant dessert in institutional cafeterias.

A healthy elderly person can consume three servings of fruit per day, ensuring a good intake of antioxidants that prevent cellular degeneration and premature aging, as well as important fibers, vitamins, and minerals for the proper functioning of the body [29]. Fruits contain compounds such as lycopene, polyphenols, and resveratrol, which help prevent prostate cancer, hypertension, and cardiovascular diseases [30].

Regarding the offering of sweets, canned fruit, or fruit preparations, in the present study, the recommendations were not met in all the evaluated menus. These results are consistent with those found by Candeias and Rego [28], who estimated an excessive offer of sweet desserts, and by Reis, Figueiredo, and Ávila [23], which observed that the “desserts” domain showed that not always only one sweet or canned fruit was provided per week, which may contribute to excessive sugar consumption. The study by Lima and Rocha [25] shows that the recommendations for offering sweets and canned fruit were met in 75% to 100% of the evaluated menus, and the research by Cardoso [16] reveals that the intake of sweets was facilitated, both at lunch and dinner, once a week on different days.

The provision of water and regular bread should be included in all meals in the menu, as they are already used, in order to enrich it. With these suggestions for actions that already exist in reality but are not included in the menus, the qualitative evaluation, for example, of Menu 2 would change from being classified as “acceptable” (68.84%) to “good” (77.32%).

The menus are prepared by personnel without specific training in the field of nutrition, such as social service technicians, sociocultural animators, nurses, and supervisors of services. Although there is occasional supervision for the inclusion of allergens in the menus by a food engineer, there is no involvement in their preparation or in the actual cooking process. The guidelines of the “Programa Nacional para a Promoção da Alimentação Saudável” of the Portuguese “Direção Geral de Saúde”, specifically those by Ferreira et al. [10], are sometimes mentioned in the support material for menu planning, but they are not always correctly implemented in practice. An evaluation of private social solidarity institutions in Matosinhos, Portugal, revealed that the majority of menus were prepared without the participation of qualified professionals, and half of them were not supported by any materials [31].

Having established criteria for various parameters of meal quality, it is necessary to ensure that they are met and monitored. This can be achieved by the involvement of specialized healthcare professionals in menu planning, as well as the availability of technical sheets for all meals served.

As a proposed improvement in menu quality, the authors suggest that everything offered to the users be included in the menu, which currently does not include all five daily meals (breakfast, lunch, afternoon snack, dinner, and supper). We would recommend the daily inclusion of raw and cooked vegetables, always prioritizing seasonal vegetables. Regarding protein sources, the presence of eggs is recommended as either the only or the main protein source, at least once a week. A reduction in the offering of sweets, canned fruit, and fruit preparations would also be advised. Seasonal fruit should be consistently included in the menus. Furthermore, the use of whole foods as additions or substitutions for more processed options would be a significant improvement in menu quality.

Appropriate nutritional monitoring should be implemented, with regular assessment of the elderly individuals’ nutritional status and adjustments to their diet and, consequently, the institution’s menu, as needed.

This evaluation should also consider the sensory and experiential aspects of food, such as taste, texture, appearance, and flavor.

It would be beneficial to identify any issues with the food served, such as a lack of flavor, unpleasant appearance, or difficulty in chewing or swallowing. Incorporating feedback from the elderly individuals themselves can help ensure that the food served meets their preferences and needs.

This tool, which is easy to apply and use, constitutes a valuable source of information for diagnosing the food offerings in various institutions, contributing to the progressive improvement of food provision and even to the enhancement and updating of guidelines in this field. This qualitative menu analysis instrument can be included in a broader strategy to improve food services, although it does not replace the need for an assessment of individual needs and tailored intervention by specialized professionals, particularly nutritionists, in each situation.

It is worth noting that the principles for menu planning defined in this tool are not naturally adapted to the various clinical situations that may occur in the elderly, so individuals with specific dietary needs require a personalized intervention approach. This tool can assist institutions when providing support to the population aged 65 or older in operationalizing the guiding rules for menu planning present in existing quality management manuals.

## 5. Conclusions

In conclusion, this study provided valuable insights into the quality of menus provided by an institution catering to the elderly population. The evaluation revealed that all the menus were classified as “acceptable” based on the assessment percentages. However, there is room for improvement, particularly in increasing the presence of eggs as a primary protein source and including seasonal fruit daily while avoiding canned fruit.

This instrument proved to be simple to use and useful for reflecting dietary frequency and the quality and content of the diet. It can be utilized by health professionals and others who are not professionals in dietary assessment research. The overall food and nutritional goal is to consume foods with the highest possible nutritional density, especially in elderly individuals who consume small quantities. The qualitative meal rating system can be used as an important link between the patient and healthcare professionals to involve the patient in their own meal planning or selection, thereby promoting healthy eating.

Moreover, the findings underscore the importance of coordinated efforts within the institution to enhance menu planning practices. This includes not only preserving aspects deemed appropriate but also addressing inadequacies to ensure the provision of nutritionally balanced and diverse meals tailored to the needs of the elderly residents.

Moving forward, it is recommended that menu planning incorporates suggestions for improvement identified in this study. Additionally, ongoing monitoring and adjustment of menus based on nutritional guidelines and feedback from the elderly individuals themselves are essential for promoting their health and well-being. Overall, this study highlights the significance of continuous evaluation and improvement in menu planning practices for elderly care institutions.

## Figures and Tables

**Table 1:** Menus 1, 2, 3, and 4 evaluation.

Domain	Item		Menu 1	Menu 2	Menu 3	Menu 4
General Items	1.1 A menu consisting of 5 daily meals.		NC	NC	NC	NC
	1.2 Exclusive offer of water, self-service, during lunch and dinner.		NC	NC	NC	NC
	1.3 Absence of monochromatic meals.		C	C	C	C
	1.4 Offer of products from regional or national production.		NC	NC	NC	NC
	1.5 Absence of dishes with similar consistency components.		C	C	C	C
	1.6 Equitable offering among various cooking methods (except frying).		C	C	C	C
	1.7 Offer of one unit of bread for lunch and dinner.		NC	NC	NC	NC
	1.8 Offer of one unit of whole grain or mixed cereal bread for lunch and dinner.		NC	NC	NC	NC
	1.9 Absence of repeated dishes within a month.		NC	NC	NC	NC
	1.10 Presence of charcuterie products up to 1 time per week.		C	C	C	C
	1.11 Absence of fried food and sweets on the same day.		C	C	C	NC
	1.12 Offer of fried foods, at most, 1 time per week.		C	C	C	C
	1.13 Absence of fried foods for dinner.		C	C	C	C
	1.14 Repetition of the same legume (in the dish or whole in the soup) not exceeding twice per week.		NC	C	C	C
	1.15 The dish composed of protein supplier, carbohydrate supplier, and vegetable accompaniment.		C	C	C	C
	1.16 Existence of technical sheets for meals.		NC	NC	NC	NC
	1.17 Use of raw potatoes in the soup preparation.		C	C	C	C
	1.18 Removal of visible skins and fats from protein suppliers before cooking.		C	C	C	C
	1.19 Offer of cooked fruit, without added sugar, a maximum of 3 times per week.		C	C	NC	C
	1.20 Daily offer of at least 3 servings of fruit (1 serving ≈ 1 medium-sized fruit).		NC	NC	NC	NC
	1.21 Absence of repeated fruit on the same day or on consecutive days.		NC	C	C	C
	1.22 Offer of at least two daily servings of milk or yogurt, preferably semi-skimmed (1 serving ≈ 240 mL) in intermediate meals.		C	C	C	C
	1.23 Offer of foods from the cereal and derivatives group in all intermediate meals (1 bread or 6–8 biscuits or a cup of low-sugar cereal).		C	C	C	C
	1.24 Cereal or derivative foods present in intermediate meals (1 bread or 6–8 biscuits or a cup of low-sugar cereal) are preferably whole.		NC	NC	NC	NC
	1.25 Equitable distribution of offerings regarding different bread accompaniments in intermediate meals.		C	C	C	C
	1.26 Inclusion of nuts with a minimum frequency of once a week (quantity equivalent to 1/3 cup of walnuts or almonds or hazelnuts or peanuts or pine nuts or 2 tablespoons of seeds), preferably for dessert or integrated into intermediate meals.		NC	NC	NC	NC
	Total	Compliance	14	16	15	15
		Non-compliance	12	10	11	11
		Item score	35	39	38	37
Soup	2.1 Offer of soup for lunch and dinner.		C	C	C	C
	2.2 Offer of dishes without vegetables, in place of soup, a maximum of once a week (broth, fish, or meat soup).		C	C	NC	C
	2.3 Offer of soup with legumes (either as a base or not) at least 3 times a week.		NC	C	C	C
	2.4 Repetition of soups up to 3 times per week, at most, and never on the same day or on consecutive days.		NC	NC	NC	C
	Total	Compliance	2	3	2	4
		Non-compliance	2	1	2	0
		Item score	4	6	5	8
Protein Suppliers	3.1 Number of fish meals equal to or greater than meat meals.		C	C	C	C
	3.2 Presence of eggs as the sole or main protein source, 1 to 2 times per week.		NC	C	NC	C
	3.3 Offer of white meats, at least 3 times per week.		C	C	C	C
	3.4 Offer of red meats a maximum of 2 times per week.		C	C	C	C
	3.5 Presence of fatty fish at least twice per week.		C	NC	C	C
	Total	Compliance	4	4	4	5
		Non-compliance	1	1	1	0
		Item score	12	11	12	14
Carbohydrate Suppliers	4.1 Equitable offering among the main carbohydrate suppliers (rice, potatoes, and pasta).		C	C	C	C
	4.2 Offering carbohydrate suppliers, with a preference for whole grains, in main meals.		NC	NC	NC	NC
	4.3 Offering legumes on the plate at least 2 times per week, as a complement or substitution for the carbohydrate source.		C	C	NC	C
	Total	Compliance	2	2	1	2
		Non-compliance	1	1	2	1
		Item score	4	4	2	4
Vegetable Suppliers	5.1 Equitable offering between cooked and raw vegetables.		C	C	C	C
	5.2 Equitable offering among seasonal vegetables.		NC	NC	NC	NC
	Total	Compliance	1	1	1	1
		Non-compliance	1	1	1	1
		Item score	2	2	2	2
Dessert	6.1 Sweet dessert or fruit in syrup, a maximum of 1 time per week.		C	C	NC	C
	6.2 Offering fresh fruit in all main meals (lunch and dinner).		NC	NC	NC	NC
	6.3 Offering fruit with a preference for seasonality.		C	C	C	C
	Total	Compliance	2	2	1	2
		Non-compliance	1	1	2	1
		Item score	4	4	1	4
Final evaluation	Quantitative (%)		60.73	68.84	62.31	63.95
	Qualitative		Acceptable	Acceptable	Acceptable	Acceptable

Legend: C—Compliance; NC—Non-compliance.

## Data Availability

Data are contained within the article.
